# A case report of maturity-onset diabetes of the young (MODY12) in a Chinese Han patient with a novel *ABCC8* gene mutation

**DOI:** 10.1097/MD.0000000000032139

**Published:** 2022-12-09

**Authors:** Yuan Wang, Chao Kang, Qiang Tong, Hui Wang, Rui Zhang, Qiao Qiao, Qian Sang, Xiaocui Wang, Jian Wang, Jing Xu

**Affiliations:** a Department of Endocrinology, Xinqiao Hospital, Army Medical University, Chongqing, China; b Department of Nutriology, The General Hospital of Western Theater Command, Chengdu, Sichuan Province, China; c Department of Nutriology, Xinqiao Hospital, Army Medical University, Chongqing, China.

**Keywords:** *ABCC8* gene, case reports, missense mutation, MODY, p.Glu1326Lys

## Abstract

Maturity onset diabetes of the young (MODY) is the most common type of monogenic diabetes, characterized by autosomal dominant inheritance, the age of onset is often <25 years old, and the clinical manifestations are atypical. MODY12 is caused by a rare missense mutation of adenosine triphosphate (ATP)-binding cassette transporter subfamily C member 8 (*ABCC8*) gene and more than 50 *ABCC8* variants were associated with MODY12.

**Patient concerns:**

The patient was a 30-year-old Chinese Han man. He was overweight with a poor control of blood glucose.

**Diagnoses:**

The patient was diagnosed with MODY12.

**Interventions:**

The patient was given glimepiride (4 mg/d) with diet and exercise therapy to reduce blood glucose and weight.

**Outcomes:**

The level of fasting blood glucose and C-peptide was improved after 1 year treatment as well as body weight.

**Lessons:**

A Chinese Han adult with a heterozygous missense mutation c.3976G > A (p.Glu1326Lys) was diagnosed with MODY12, which was the new pathogenic mutation for the disease. This report expands the spectrum of variants causing MODY12 and reduces misdiagnosis.

## 1. Introduction

Maturity onset diabetes of the young (MODY) comprises a group of heterogeneous monogenic disorders. There are 14 kinds of MODY pathogenic genes which have been discovered and they account for about 1 to 2% of all diabetic patients.^[1,2]^ The subtypes of MODY vary in clinical features, prevalence and treatment.^[3]^ The MODY subtypes 1 to 5 are characterized the best, with MODY2 and MODY3 accounting for 90% of all MODY cases.^[4]^ MODY12 is caused by a pathogenic mutation which can be found in the adenosine triphosphate (ATP)-binding cassette transporter subfamily C member 8 (*ABCC8*) gene, located on chromosome 11, encoding sulfonylurea receptor 1 (SUR1), and the incidence of MODY12 is accounting for about 1% of the entire MODY.^[5]^ The *ABCC8* gene consists of 39 exons encoding for 1582 amino acids. The phenotype of an *ABCC8* mutation can be varying, and the genetic spectrum is equally complex. It has been reported that 55 *ABCC8* variants were associated with MODY12 up to date.^[6]^ In this article, we present a case of a 30-year-old Chinese Han man in whom diabetes was diagnosed with a new c.3976G > A (p.Glu1326Lys) *ABCC8* variant causing MODY12.

## 2. Methods

### 2.1. Personal and family history

**Figure 1.**
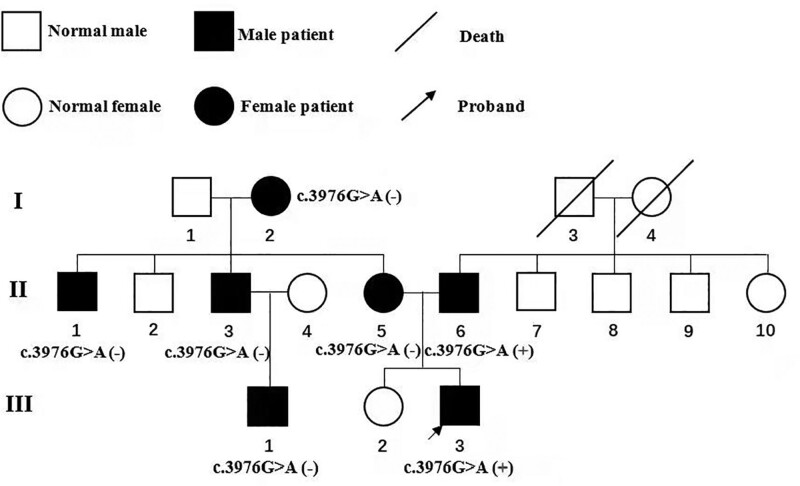
Pedigree of family with diabetes.

The proband was a 30-year-old Chinese Han man. He was found for fasting blood glucose as high as 10.0 mmol/L without xerostomia, polydipsia, polyuria, polyphagia, and weight loss 6 years ago. The patient was diagnosed with diabetes, and diet and exercise therapy were given to control his high level of blood glucose. Self-monitoring blood glucose was taken and his blood sugar could be controlled within the normal range. Five years ago, his fasting blood sugar levels were risen again. According to the level of C-peptide, the local hospital diagnosed him with type 2 diabetes and metformin combined with Gansulin 30R twice a day was the treatment protocols for him. However, he reported that his blood glucose was still fluctuating, but he gave up further treatment. Two years ago, he found that the glycosylated hemoglobin (HbA1c) was 9.6% and fasting C-peptide was 1.81 ng/mL during his physical examination. The dose both of metformin and Gansulin 30R were increased for treatment. Once again, his blood sugar was not well-controlled and he went to our hospital for further treatment. He had no history of smoking or drinking. The family contained 3 generations of diabetics (Fig. 1). A total of 7 people have been diagnosed with diabetes or impaired glucose tolerance.

### 2.2. Data analysis

Deoxyribonucleic acid (DNA) was extracted from the venous blood by using the QIAamp DNA Blood Midi kit (Qiagen, Germany). Then the qualified DNA was examined by whole-exome sequencing (WES) approach using xGen Exome Research Panel (IDT). The MODY-related genes (*ABCC8, AKT2, BLK, CEL, EIF2AK3, GCK, GLIS3, GLUD1, HADH, HNF1A, HNF1B, HNF4A, INS, INSR, KCNJ11, KLF11, MAPK8IP1, NEUROD1, PAX4, PDX1, PLAGL1, PTF1A, RFX6, SLC19A2, SLC2A2, UCP2* and *ZFP57*) were detected through Illumina NovaSeq 6000. Mean sequencing depth and nucleotides with >20x sequencing depth were 269x and 99.93%, respectively. The raw sequencing reads were aligned by using the Burrows–Wheeler Aligner (BWA). The pathogenicity of the variant was classified according to the established guidelines of the american college of medical genetics and genomics (ACMG) and association for molecular pathology (AMP).^[7]^ It was analyzed to predict mutational effect with the following software: Annovar,^[8]^ CADD,^[9]^ Sift,^[10]^ Polyphen2,^[11]^ MutAssesor,^[12]^ Fathmm^[13]^ and VEST.^[14]^ Regarding the effect assessed by the predictors, we assigned each variant a prediction score with a maximum of 7 points following the criteria: 1 = “Damaging/High,” 0.5 = “Possibly damaging/Moderate” and 0 = “Benign,” the score was used to sum up the level of evidence from the predictors in order to classify the variants following the ACMG guidelines. To illustrate the effects of the missense mutation in exons, a 3-dimensional structure was constructed using SWISS-MODEL (https://swissmodel.expasy.org/). Structural graphics and visualization were exhibited by PyMOL software.

## 3. Results

### 3.1. Physical examination

Blood pressure 122/84 mm Hg, height 170 cm, weight 76 kg, body mass index 26.30 kg/m^2^, HbA1c 9.0%, uric acid (UA) 488.4 μmol/L, alanine aminotransferase (ALT) 75.0 IU/L, triglyceride (TG) 3.21 mmol/L, total cholesterol (TCH) 5.37 mmol/L, low-density lipoprotein cholesterol (LDL-C) 2.67 mmol/L; fasting C-peptide 1.38 ng/mL, C-peptide 30 minutes 1.90 ng/mL, C-peptide 60 minutes 2.50 ng/mL, C-peptide 120 minutes 2.48 ng/mL, C-peptide 180 minutes 2.33 ng/mL. Diabetes autoantibodies (glutamate decarboxylase, insulin antibodies, and pancreatic islet cell antibodies) were negative. Cardiac ultrasound showed that tricuspid valve regurgitation. The optic fundi revealed no hemorrhages or exudates, and the fundal vessels were unremarkable. Neuroelectromyography was normal.

### 3.2. Exome sequencing in a family with MODY12 identifies a new coding variant in *ABCC8*

**Figure 2.**
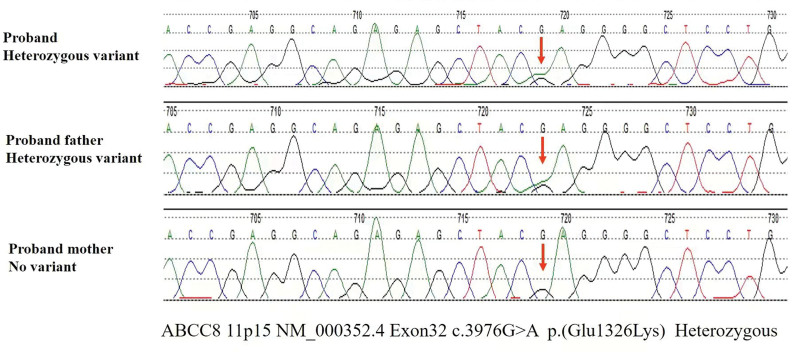
Gene sequencing results of proband and his family.

**Figure 3.**
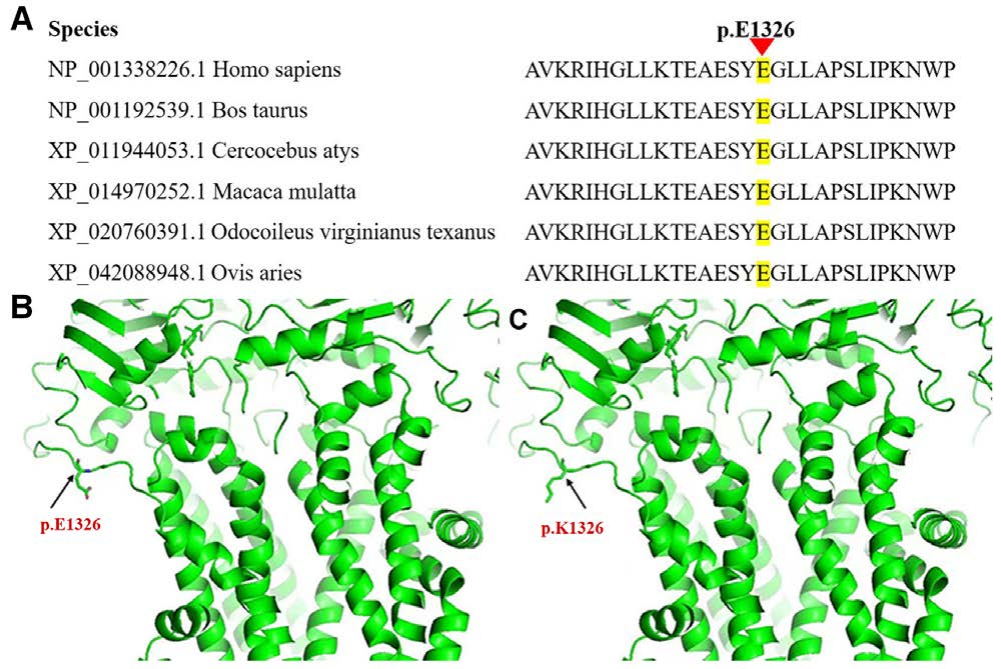
(A) Amino acid sequence alignment of the mutation c.3976G > A. (B) Molecular model of the wild-type (C) in comparison with the missense mutation p.E1326K. E/Glu = glutamic acid; K/Lys = lysine.

The patient was highly suspected as MODY under diagnostic criteria. MODY was severely depended on genetic diagnosis. After obtaining informed consent, a venous blood was collected from the proband and all his family members except those who have passed away (I3 and I4, Fig. 1). The exome sequencing data suggested that the proband has a heterozygous mutation in exon 32 of the *ABCC8* gene, c.3976G > A (p.Glu1326Lys) (Fig. 2). No mutations were detected for the remaining genes. Amino acid sequence alignment revealed that this mutation c.3976G > A (p.Glu1326Lys) was in the conserved domain by using BLASTp in national center for biotechnology information (NCBI) (Fig. 3A). The protein structure of this mutation c.3976G > A (p.Glu1326Lys) was visualized by using PyMOL software (Fig. 3B and C). The result of the variant in silico analysis was as follows: Annovar = Moderate, CADD = Damaging, Sift = Possibly damaging, Polyphen2 = Benign, MutAssesor = Benign, Fathmm = Damaging, and VEST = Damaging. The prediction score was 4 and the ACMG classification was likely pathogenic. Combined with the patient’s condition and the treatment effect, the protein structural changes caused by this mutation may ultimately lead to the occurrence of the disease. His father was a heterozygous carrier. Other family members did not carry this mutant gene. The father was newly diagnosed with diabetes despite of having no history of diabetes before this examination. The patient was finally diagnosed with MODY12 according to WES.

### 3.3. Treatment and follow-up

**Figure 4.**
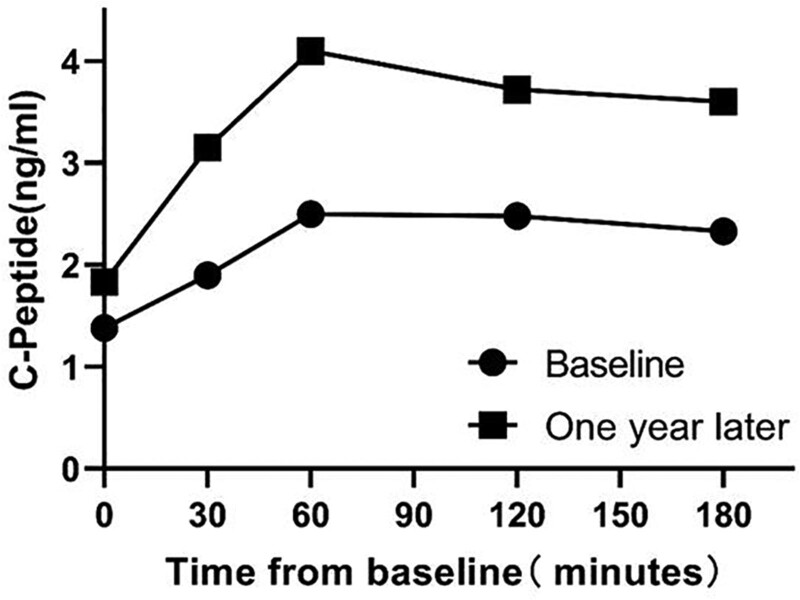
C-peptide of the patient.

The patient was given glimepiride (4 mg/d) with diet and exercise therapy after being diagnosed with MODY12. One year later, he was 73 kg with body mass index 26.30 kg/m^2^. Fasting blood glucose was 7.2 mmol/L and HbA1c was 6.8%. We once again perfected the C-peptide release assay, and the result was shown in Figure 4. The level of C-peptide was improved than 1 year before.

## 4. Discussion

We reported a case of Chinese Han adult diagnosed with MODY12. A new heterozygous site mutation c.3976G > A (p.Glu1326Lys) of *ABCC8* was reported, providing a new mechanism of MODY12. Pathogenic mutation of *ABCC8* is associated with MODY12. *ABCC8* is located in the ATP-binding domain of 11p15.1.^[15]^ Previous study reported that 7 patients had *ABCC8* mutant genes with diverse clinical manifestations, of which 4 were new mutation types: E100K, G214R, Q485R, N1245D. *ABCC8* has been cloned in 1999, which encodes the sulfonylurea receptor of the KATP channel.^[16]^ It affects the potential changes of the β cell membrane to deteriorate the function of KATP channel, which could reduce the level of insulin secretion. Bowman first reported MODY12 in 2012 and found that 7 patients had *ABCC8* mutant genes with diverse clinical manifestations, of which 4 were new mutation types: E100K, G214R, Q485R and N1245D.^[15]^ We found a Chinese Han adult with a heterozygous missense mutation c.3976G > A (p.Glu1326Lys) who was diagnosed with MODY12, which was the new pathogenic mutation for the disease.

To date, there are few large-scale clinical studies on MODY12, most of which are case reports. There are currently >50 cases reported.^[17]^ Tarasov reported 3 cases of Y356C *ABCC8* mutations, which caused insulin deficiency and high fasting blood glucose.^[18]^ A missense mutation of *ABCC8* gene was found in a 39-year-old French patient with normal weight and hyperglycemia, which tyrosine replaces cysteine (SUR1Y356C). In recent years, with the wide application of gene sequencing, many countries gradually began to study the gene variants of MODY. The prevalence of MODY12 is very low. In Turkey, mutation analysis of MODY genes was performed in 106 patients with a clinical diagnosis of MODY.^[19]^ A total of 18 variants were revealed among all patients. Most of the variants were identified in the glucokinase gene, and the frequency was only 6% (1/18) for *ABCC8* genes. Also, 102 Brazilians who with clinical suspicion of MODY had undergone targeted sequencing and 7 variants of *ABCC8* were found.^[20]^ In Korea, 40 patients with suspected monogenic diabetes who had more than 50% of MODY probability were tested. A total of 7 variants including 2 variants of *ABCC8* were identified.^[21]^ Recently, a study in UK found that the mitochondrial m.3243A > G and mutations in HNF1B were responsible for the majority of mutations in syndromic diabetes genes, and 11 patients, about 4% in 1280 patients, were found the variant for ABCC8.^[22]^ However, a study carried on South India showed HNF1A and *ABCC8* to be the most frequently mutated MODY genes, with 7.2% (11/152) and 3.3% (5/152) respectively.^[23]^

Nowadays, MODY has been given more attention in China. A MODY12 family recently reported that the proband was a 12-year-old boy with ketosis at onset, but the C-peptide level was high. He was confirmed as MODY12 through genetic diagnosis and treatment for metformin.^[24]^ In a cross-sectional study on early-onset diabetes in China, a total of 543 patients were enrolled, and it was finally found that 8 patients had *ABCC8* gene mutations, with an incidence rate of about 1.5%.^[25]^ There is also a recent report about MODY12 in children. The proband had a c.3976G > A heterozygous mutation in the 32nd exon of *ABCC8* gene, which is the same position of our case. The gene mutation was adopted from his mother, but her mother did not have diabetes.^[26]^ So, whether the c.3976G > A variant of the *ABCC8* gene is the cause of the disease in this patient or not depends on the further functional studies and more case data. The c.3976G > A variant of the *ABCC8* gene had been presented in 1KGenome (7 East Asian, only 1 is man) and in the GnomAD3.1 controls (15 allele counts among 5184 in East Asian, 3 men). This variant was previously reported as a cause of permanent neonatal diabetes and pulmonary arterial hypertension,^[27,28]^ but not as a pathopoiesia for MODY12.

In our case, genetic testing revealed that the father of the patient was a heterozygous carrier, but the father had no history of diabetes. The mother and grandmother with a history of diabetes seemed to be inconsistent with the law of inheritance, but then the father was diagnosed with diabetes by oral glucose tolerance test (OGTT), and the patient was finally diagnosed with MODY12. If the patient’s mother and grandmother do not have diabetes, this patient is likely to be misdiagnosed. Therefore, the clinical manifestations of MODY are diverse, medical history needs to be carefully recorded, especially for young patients.

The *ABCC8* gene mutation of MODY12 is complex and diverse. Because sulfonylureas can specifically bind to the sulfonylurea receptor 1 (SUR1) subunit, close the channel in an ATP-independent manner and release insulin.^[29]^ Therefore, MODY12 should be sensitive to oral sulfonylureas. Previous studies have found that all carriers of ABCC8 mutations could be switched to sulfonylureas, and this could achieve a reduction in HbA1c.^[30]^ In this case, the initial dose of glimepiride was 4 mg/day to avoid hypoglycemia. Besides, the patient received exercise and dietary therapy at the same time. The HbA1c was 6.8% when he was discharged, and the dose of glimepiride was not changed. The patient was pleased with the outcome.

Here, the clinical manifestations of MODY12 in this case are not unique. Making use of insulin therapy may bring about the risk of weight gain in the future. It is easy to be misdiagnosed as type 2 diabetes based on the medical history and auxiliary examinations. Although the patient’s mother and grandmother have diabetes, the mother does not carry the disease-curing gene. Therefore, genetic diagnosis plays an important role in the diagnosis of MODY12. This case reported a heterozygous mutation in MODY12 which is newly found in Chinese Han adults, indicating the value of genetic testing in young patients.

## Author contributions

JW and JX designed the experiments. YW, QT, HW, RZ, QQ, QS and XW collected samples and performed the experiments. YW, CK and JX analyzed the data. JX prepared the manuscript and had primary responsibility for final content. All authors read and approved the final manuscript.

**Data curation:** Hui Wang.

**Formal analysis:** Rui Zhang.

**Investigation:** Yuan Wang, Chao Kang, Qiang Tong, Qian Sang.

**Methodology:** Qian Sang, Xiaocui Wang.

**Project administration:** Jing Xu.

**Supervision:** Jian Wang.

**Visualization:** Qiao Qiao.

**Writing – original draft:** Yuan Wang, Chao Kang.

**Writing – review & editing:** Jian Wang, Jing Xu.
